# Automated Alphabet Reduction for Protein Datasets

**DOI:** 10.1186/1471-2105-10-6

**Published:** 2009-01-06

**Authors:** Jaume Bacardit, Michael Stout, Jonathan D Hirst, Alfonso Valencia, Robert E Smith, Natalio Krasnogor

**Affiliations:** 1 ASAP research group, School of Computer Science, University of Nottingham, Jubilee Campus, Wollaton Road, Nottingham, NG8 1BB, UK; 2 MYCIB, School of Biosciences, University of Nottingham, Sutton Bonington, LE12 5RD, UK; 3 School of Chemistry, University of Nottingham, University Park, Nottingham, NG7 2RD, UK; 4 Spanish National Cancer Research Centre, Melchor Fdez Almagro, 3. 28029 Madrid, Spain; 5 Dept. of Computer Science, University College London, Gower Street, London, WC1E 6BT, UK

## Abstract

**Background:**

We investigate automated and generic alphabet reduction techniques for protein structure prediction datasets. Reducing alphabet cardinality without losing key biochemical information opens the door to potentially faster machine learning, data mining and optimization applications in structural bioinformatics. Furthermore, reduced but informative alphabets often result in, e.g., more compact and human-friendly classification/clustering rules. In this paper we propose a robust and sophisticated alphabet reduction protocol based on mutual information and state-of-the-art optimization techniques.

**Results:**

We applied this protocol to the prediction of two protein structural features: contact number and relative solvent accessibility. For both features we generated alphabets of two, three, four and five letters. The five-letter alphabets gave prediction accuracies statistically similar to that obtained using the full amino acid alphabet. Moreover, the automatically designed alphabets were compared against other reduced alphabets taken from the literature or human-designed, outperforming them. The differences between our alphabets and the alphabets taken from the literature were quantitatively analyzed. All the above process had been performed using a primary sequence representation of proteins. As a final experiment, we extrapolated the obtained five-letter alphabet to reduce a, much richer, protein representation based on evolutionary information for the prediction of the same two features. Again, the performance gap between the full representation and the reduced representation was small, showing that the results of our automated alphabet reduction protocol, even if they were obtained using a simple representation, are also able to capture the crucial information needed for state-of-the-art protein representations.

**Conclusion:**

Our automated alphabet reduction protocol generates competent reduced alphabets tailored specifically for a variety of protein datasets. This process is done without any domain knowledge, using information theory metrics instead. The reduced alphabets contain some unexpected (but sound) groups of amino acids, thus suggesting new ways of interpreting the data.

## Background

The prediction of the 3D structure of protein chains, known as Protein Structure Prediction (PSP), is a key challenge in structural bioinformatics. Not only there is a lack of consensus on how to approach PSP, but for some of the current methods, especially ab-initio ones, computations are exceedingly demanding. Rosetta@home [1], one of the top predictors in the CASP7 (Critical Assessment of techniques for protein Structure Prediction) experiment, used up to 10000 computing days to model a single protein. One way in which PSP calculations might be accelerated is by using a divide-and-conquer approach, where the problem of predicting the tertiary structure of a given sequence is split into smaller challenges, such as predicting secondary structure, solvent accessibility, coordination number, etc. and then using the solutions for these simpler problems as constraints, i.e. building blocks, for the original 3D prediction. A complementary strategy would be to reduce the size of the alphabet of the variables that are involved in the prediction of tertiary structure, and solve the previously mentioned sub-problems using the reduced alphabets. The alphabet by which the sequence of a protein is represented would be an obvious focus for any reduction mechanism.

An example of a widely used reduction is the two-letter hydrophobic/polar (HP) alphabet [2]. This reduction is usually followed by constraining the residue locations of the protein to those of a 2D/3D lattice [3,4]. Sometimes this HP alphabet reduction is applied to off-lattice proteins. For example, in a recent paper [5] we compared predictions of residue contact numbers (CN) for lattice and off-lattice proteins using both the HP alphabet and the amino acid (AA) alphabet. The HP alphabet gave predictions significantly less accurate than the AA alphabet, although the difference was not large (at most 3.8%). The reduction of alphabets for PSP related problems brings to the fore some important questions. (1) Is it possible to reduce alphabet size without significantly losing information and hence degrading performance? (2) Is there a single reduced alphabet for all problems? Or would, for example, the prediction of disulfide bonds necessitate a different alphabet than, for instance, the prediction of contact numbers?

### Previous work

The alphabet reduction problem in the context of PSP can be summarized in a simple question: which is the minimum number of AAs that is able to represent the structural information of a protein? That is, when is the loss of information intrinsic in the alphabet reduction problem going to affect crucial data? In general this kind of process involves deciding on three aspects: (1) how are we going to represent the protein structural information? (2) how are we going to quantify how good is the reduced alphabet to maintain the crucial information held in the representation from point 1 and (3) how are we going to obtain the optimal reduced alphabet based on the metric of point 2.

The work of Solis and Rackovsky [6] is one of the earliest alphabet reduction methods that, like our approach, uses information theory to quantify the quality of the reduced alphabets. They propose an information gain function that measures how different the entropy of the structural patterns represented by a given reduced alphabet is from a random distribution of patterns, and then optimize this function using a Monte Carlo method to obtain their optimal reduced alphabet. Two representations of a protein structure were evaluated in that work, one based on bond lengths, bond angles and dihedral angles of the protein's backbone and another one based on secondary structure states of the protein residues. Other work of these authors combining information theory and reduced alphabets includes [7] and [8]. The latter one also introduces the simultaneous generation of multiple reduced alphabets for different parts of the protein representation, which is similar to our DualRMI strategy.

Other methods [9,10] base their metrics on the Miyazawa-Jernigan interaction matrix [11], while others [12] are based on the BLOSUM substitution matrix [13]. Another work [14] uses the Kullback-Leibler distance [15] between probability distributions as their metric to evaluate reduced alphabets, applied to a four-state secondary structure representation. One example of the application of an Evolutionary Algorithm to alphabet reduction is [16]. In this case, a genetic algorithm (GA) was used to optimize a five-letter alphabet for sequence alignment. The GA was used to maximize the difference between the sequence identity of the training alignments and a set of random alignments based on the same sequences. Another approach [17] applied Mutual Information to optimize alphabet reduction for contact potentials. Meiler et al. [18] proposed a method whereby, instead of treating each AA type at a symbolic level, they were characterized through several physical features and a neural network was applied to generate a lower dimensionality representation of these features. Wrabl and Grishin [19] used the Monte Carlo method to optimize groups of amino acids that maximize the total variance of amino acid frequencies applied to multiple sequence alignments. A different structural representation was used by [20], where the 3D structure of a protein is mapped into protein blocks of five residues long containing eight consecutive (*ψ*, *φ*) dihedral angles. The distributions of AAs in the protein blocks were used to group together those having similar local structure.

Reduced alphabets for protein sequences have other traditional uses besides PSP, such as protein design and mutation, where the goal is to obtain sequences, either generating them from scratch or modifying previously known proteins, that fold in a certain specific way or to obtain/maintain certain functional properties. To that end, it is important to known which AAs can be exchanged with which others without impacting in the fold or the function/stability/etc. of the protein. That is, which AAs can be grouped together because they behave similarly. A classic example of this kind of research is [21], where several proteins with a certain specific alternation of polar/nonpolar residues were designed and all of them folded into four helix bundle proteins. The specific AA sequence of each protein varied, but the pattern of polar/nonpolar residues was always the same. Another example is [22], where an enzyme used for D-amino acids production was randomly mutated, and the individual AA substitutions that maintained the enzyme function and increased its thermostability were identified. In [23] a 213-residue E. Coli enzyme was mutated with the objective of obtaining a functionally similar protein having a reduced set of amino acids. In the final variant of the enzyme, after 73 substitutions, nine AA types occupied 88% of its sequence, and seven AA types were never present.

In recent work [24] we proposed an automated method to perform alphabet reduction. This method uses ECGA [25] to optimize the distribution of the 20 letters of the AA alphabet into a predefined number of categories, using the Mutual Information (MI) metric, as an objective function. This measure relates the dependence between two variables: the input attributes and the feature we are predicting. By optimizing this measure we are looking for the alphabet reduction that maintains as much of the useful information as possible existing in the input attributes related to the predicted feature. We applied this measure to predict CN [26], optimizing alphabets ranging from two to five letters. Afterwards, the dataset with reduced representation was fed into the BioHEL machine learning method [27] to determine if the reduced alphabet was able to perform competently. It was possible to obtain an alphabet of three letters with similar performance to the full AA alphabet using a protein-wise accuracy metric.

However, when the sample size was small, e.g. trying to optimize alphabets with more than three symbols, the MI was unable to find a reduced alphabet without losing performance. Table 1 contains, for each tested alphabet size in our previous work, the percentage of possible input patterns actually represented in the dataset. We were predicting the CN of a residue using as input information the AA type of a window of ± 4 residues around the target. Therefore, if a two-letter alphabet was used to represent these data, there would be a total of 512 possible input patterns (two letters and nine window positions). If we were using a three letters alphabet, we would have 19683 possible input patterns. As the training set contained around 230000 instances, with two and three letters it was (almost) possible to have at least one occurrence of all possible input patterns. However, beyond three letters only a fraction of the input patterns is available, and mostly with a single instance per pattern. An extreme case that illustrates the small sample size issue is if we were to compute this percentage of represented patterns for the original 20-letter AA alphabet. The difference between the size of the, already large, training set and the size of the input space is many orders of magnitude. MI needs *redundancy *in order to estimate properly the relation between inputs and outputs, and there is almost no redundancy in the dataset for alphabets with more than three letters. This objective function cannot provide appropriate information for a successful alphabet optimization. Our experiments confirmed a performance degradation for alphabets of four and five letters and, moreover, it was difficult to extract meaningful explanations for the resulting reduced alphabets. In this paper we use an existing method [28] to increase the robustness of the MI metric, which is implemented into two of our alphabet reduction strategies, RMI and DualRMI (defined in next section).

**Table 1:** Percentage of input space covered by training instances for various alphabet sizes (CN feature)

# letters	Ratio
2	100%
3	97.8%
4	57.6%
5	11.3%
20	3.1*e*^-7^

### Our contribution

In this paper, we address these issues by extending our previous work [24]. Given a dataset and a feature to predict (structural representation) one may ask "what is the optimal alphabet that must be used to represent and exploit the available data?" This is, in essence, an optimization problem for which, as stated above, a suitable objective function and optimization algorithm must be found. In this paper we propose that an existing robust MI estimation method [28] is a very strong candidate for a good objective function, while a state-of-the-art evolutionary algorithm, ECGA [25], is used to explore the vast and complex search space associated with alphabet minimization. We test our protocol on two structural bioinformatics problems, namely, contact number (CN) and relative solvent accessibility (RSA) prediction. That is, we used the automatically reduced alphabets to predict CN and RSA profiles for proteins and compared their accuracy against those of the full alphabet, reduced alphabets found in the literature [16] and with some expert-designed ones. Our results indicate that we can obtain reduced alphabets of only five letters that give an accuracy within 1% of that obtained with the full AA alphabet, and higher accuracy than the other reduced alphabets included in the comparison. The differences between the reduced alphabets are quantitatively analyzed. As a final experiment to show the generality and scientific relevance of this work, we used five-letter alphabet obtained with our protocol to reduce a protein representation using evolutionary information, namely a position-specific scoring matrix (PSSM) [29] representation, and then we repeated the process of learning from both the reduced and the original representation and compare their performance.

## Methods

### Dataset and predicted PSP features

#### Contact Number

The CN of a certain AA is a specific feature of a protein's 3D structure. That is, in the native state, each residue will have a set of other residues that are its spatial nearest neighbours. The number of nearest neighbours of a given residue is its contact number. A variety of machine learning paradigms have been used to predict this feature [26,30,31]. The CN definition we have used is the one proposed by Kinjo et al. [30]. It is defined using the *C*_*β *_atom (*C*_*α *_for glycine) of the residues. The boundary of the sphere around a residue, defined by the distance cutoff *d*_*c *_∈ ℜ^+^, is made smooth by using a sigmoid function. A minimum chain separation of two residues is required. Formally, the CN, $N_{i}^{p}$, of residue *i *in protein chain *p *is computed as:

(1) $$N_{i}^{p}=\sum_{j:\left|j-i\right|>2}\frac{1}{1+exp(w(r_{ij}-d_{c}))}$$

where *r*_*ij *_is the Euclidean distance between the *C*_*β *_atoms of the *i*th and *j*th residues. The constant *w *determines the sharpness of the boundary of the sphere. In this paper we used a distance cutoff *d*_*c *_of 10 Å and a *w *of 3. The real-valued definition of CN has been discretized in order to transform the dataset into a classification problem that can be mined by machine learning methods. We divide the range of CN values into two states (low/high CN) by cutting the CN range by its middle point. To predict the CN of each residue in a protein we use the set of input attributes labelled as *CN1 *in [26]: the input data consist of the AA type of the residues in a window of four residues at each side of the target.

In the final experiment using the PSSM representation, this information was computed by using the PSI-BLAST program [32] following the procedure suggested in [33]. Each residue in the chain is represented by 20 continuous variables. We use the same window size of ± 4 residues. Thus, each instance has 180 attributes.

#### Relative Solvent Accessibility

Another interesting PSP feature is the solvent accessibility (SA) of residues. Prediction of this feature is usually addressed after a certain AA-wise normalization, where the SA of a residue is divided by the maximum accessible surface in the extended conformation of its AA type in what is known as relative solvent accessibility (RSA) [34]. In order to predict this continuous feature some works use regression methods [35] while other works predict whether the RSA of a residue is, for instance, lower/higher than some threshold (Buried/Exposed), treating this prediction as a classification problem [34,36]. We have used the DSSP program [37] to obtain the actual SA of each residue in the dataset. Next, we compute the RSA by dividing the SA of each residue by the maximum SA values specified in [34] for each AA type. The continuous RSA was transformed into a classification problem, by dividing the domain into buried/exposed states, placing a cut point at 25% RSA, as in [36]. The input data used to predict RSA are analogous to the data used for the CN dataset: the AA type of the residues in a window of ± 4 residues around the target. The PSSM representation for RSA is also equivalent to the one used for CN: 180 continuous attributes.

#### Protein dataset

We have used the dataset and training/test partitions proposed by Kinjo et al. [30]. The protein chains were selected from PDB-REPRDB [38] with the following conditions: less than 30% of sequence identity, sequence length greater than 50, no membrane proteins, no nonstandard residues, no chain breaks, resolution better than 2 Å and a crystallographic *R *factor better than 20%. Chains that had no entry in the HSSP database [39] were discarded. The final dataset contains 1050 protein chains and 257560 residues. Data were partitioned into training and test sets using an iterated hold-out procedure (very close to the standard stratified ten-fold cross-validation procedure). The proteins included in training/test pair of sets are reported in . The original and alphabet-reduced datasets for both CN and RSA prediction and all the training/test partitions are available at  (368 MB).

### Automated Alphabet Reduction protocol

The experimental protocol follows two main stages. In the first one, ECGA is used to optimize a reduced alphabet. MI is used as the objective function. In the second stage, BioHEL [27] is used to validate the reliability of the optimized alphabet found in the first stage by training classifiers, i.e. predictors, for CN and RSA. The following protocol was used for our experiments. For each dataset (CN and RSA) and for each tested alphabet size (from two to five letters) ECGA is used to find the optimal alphabet reduction of the predetermined size using the MI-based objective function. Three MI strategies are used: MI, RMI and DualRMI (explained in the next subsection). Next, the alphabet reduction policy is applied to the dataset. Finally, the BioHEL classification method is applied to the dataset with the optimally reduced alphabet found in the previous stage. We have tested alphabets of up to five letters only, because we are (1) interested in determining the smallest possible alphabet size that suffers from only a marginal information loss and (2) because recent literature focuses on alphabets of similar sizes [16].

### Optimization of reduced alphabets

ECGA [25] is an optimization method belonging to the family of Estimation of Distribution Algorithms (EDA) [40]. EDAs are Evolutionary Computation Techniques that employ statistical learning or machine learning methods to estimate the structure of the problem, and employ these estimations to perform an intelligent exploration of the search space. In the ECGA, the structure of the population is modelled as a set of non-overlapped groups of variables. The variables in each group interact strongly among themselves, and interact less with variables in other groups. Next, an exploration mechanism seeks new solutions, exploiting the estimated problem structure model. We have extended a public implementation of ECGA  to optimize reduced alphabets. The source code of the program is available from .

#### Objective function

The aim of the alphabet reduction optimization is to simplify the representation of the dataset with the goals of (1) making the problem easier to learn and (2) enhancing the interpretability of the resulting classifiers. These two goals are, of course, counterbalanced with the need to maintain the essential information contained in the original dataset. Therefore, the objective function for such a process should give an estimation of what the reduced input information can infer about the output.

MI is a measure that quantifies how much information one variable holds about the other [41]. It is defined as:

(2) $$I(X;Y)=\sum_{y\in Y}\sum_{x\in X}p(x,y)log\frac{p(x,y)}{p(x)p(y)}$$

where *p*(*x*) and *p*(*y*) are, respectively, the probabilities of appearance of *x *and *y*, and *p*(*x*, *y*) is the probability of having *x*, *y *at the same time in the dataset. In our case, we use MI to measure the quantity of information that the input variables of the alphabet-reduced dataset have in relation to the states in which the protein structural feature is partitioned. That is, for a given instance, *x *represents a string that concatenates the input variables of the instance, while *y *encodes the associated class for that instance.

#### Alphabet reduction strategies

We describe next the three studied reduction strategies. The first corresponds to the strategy tested in our previous work [24], while the latter two represent substantial improvements.

#### Mutual Information strategy

This strategy is composed of a representation of the reduced alphabet and an objective function that evaluates such reduction. The representation is simple: it has one variable for each letter of the original alphabet (the 20 AA types plus the end-of-chain symbol) encoding the group of letters where this AA type is assigned. This variable can take a value in the range 0..*N *- 1, where *N *is the predefined number of symbols of the reduced alphabet. Table 2 illustrates an example of such encoding for a reduction process into two groups. The objective function that evaluates the reduction is the original MI metric as per eq. 2.

**Table 2:** Representation used in the alphabet reduction process for a two-letter reduction

**Orig. Alphabet**	ACDEFGHIKLMNPQRSTVWXY
**Encoding**	001100001001111110010
**Meaning of the encoding**	Group 1: *ACFGHILMV WY*
	Group 2: *DEKNPQRSTX*

Each objective function computation follows these steps: first, the reduction mappings are extracted from the representation. Next, the instances of the training set are transformed into the low cardinality alphabet based on the extracted mappings creating a set of pairs (*x*_1_, *y*_1_), (*x*_2_, *y*_2_), ..., (*x*_*n*_, *y*_*n*_). Then, MI is computed from the data, *MI *= *I*(*X*, *Y*). The objective function of this alphabet reduction is its dataset Mutual Information.

#### Robust Mutual Information

As discussed in the introduction, the performance of MI as a good objective function degrades when applied to small samples. Hence, we also use the approach proposed in [28]. The strategy is known as Robust Mutual Information (RMI). It uses the same representation encoding as in the MI strategy, but a different objective function, that is computed as follows: first, the reduction mappings are extracted from the representation and the MI measure is computed as in the previous strategy. Next, we scramble the pairs of (*x*_*i*_, *y*_*i*_), *i *∈ 1..*n *joining randomly some *x*_*i *_with some *y*_*j*_, but maintaining all *X *and *Y *from the original dataset. The shuffling process is repeated *N *times with different random seeds, and the MI measures computed from each shuffling are averaged. $MI_{s}=\frac{1}{N}\sum_{i=1}^{N}PermMI_{i}$. Finally, the objective function of this alphabet reduction is the dataset MI minus the average shuffled MI: *obj func *= *MI *- *MI*_*s*_. Intuitively, *MI*_*s *_is an estimation of the sampling bias existing in the data, unrelated to the relationship between X and Y. By removing it from the MI, we obtain a better estimation of the amount of information that *X *and *Y *share.

#### Dual Robust Mutual Information

The third reduction strategy is motivated by our findings [26], that suggested that the target AA and its environment (the window composed of the nearest neighbours of the target in the protein chain) might benefit from two *different *reductions. This observation leads us to think that it might be sensible to test the performance of a dual alphabet reduction: one reduction policy specifically for the target residue and another reduction policy for the other residues in the window. This strategy will be used in combination with RMI with the name Dual Robust Mutual Information (DualRMI) strategy.

### Verification of the reduced alphabets

To verify the results of the first stage of our protocol, we use the BioHEL machine learning method to learn the dataset with reduced representation. BioHEL (Bioinformatics-oriented Hierarchical Evolutionary Learning) is an Evolutionary-Computation based Machine Learning system following the Iterative Rule Learning approach [42]. BioHEL is also strongly influenced by GAssist [43] which is a Pittsburgh GBML system. The system applies a generational Genetic Algorithm (GA) with elitism, which evolves individuals that are classification rules. Rules are obtained by an iterative process. After each rule is obtained, the training examples that are covered by this rule are removed from the training set, to force the GA at the next iteration to explore other areas of the classification space. The performance of BioHEL is enhanced by running it several times with different initial random seeds on the same data. Afterwards, the rule sets obtained by each run are combined to form an ensemble that generates consensus prediction using a simple majority vote. For specific details of the design and objective function of BioHEL, see [27]. The source code of the program is available from .

## Results and discussion

### Contact Number prediction

**Figure 1:**
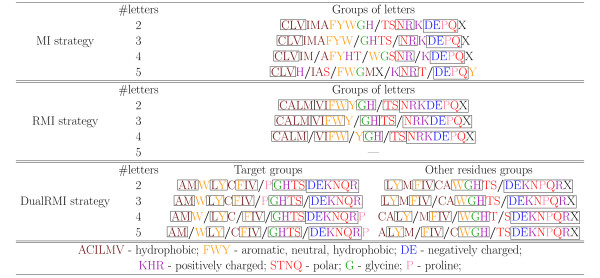
**Alphabet reductions for the CN feature**. Groups are separated by '/'. Solid rectangle marks amino acids that remain in the same group for all four alphabets.

ECGA was used to find alphabet reductions into alphabets of two, three, four and five symbols following the MI, RMI and Dual RMI strategies. Figure 1 shows the reductions obtained; for visualization of the physico-chemical properties of the AA groups obtained, we have colored each AA type differently according to the properties discussed in [44]. We have aligned as much as possible the amino acids between the reduction groups of increasing alphabet size. This allows us to observe how the optimization method rearranges the groups when the alphabet size grows. Also, we have marked with a solid rectangle the amino acids that remain within the same group with at least one other amino acid for all the four tested alphabet sizes. For simplicity, we show only the reductions obtained from the first of the ten training sets. The reduction groups for the other training sets are reported in Additional File 1. These reduction groups, although slightly different for each training set (especially in the MI strategy, quite noisy because of the small sample issue explained in the background section), provide similar statistical and predictive properties.

When ECGA is used with the RMI strategy to search the space of possible reduced alphabets with the goal of designing a five letters alphabet, it does not find a solution with five letters and reports instead a candidate alphabet with four letters. DualRMI did find a five-letter alphabet for the *target *residue, but only a four-letter alphabet for the other residues. Thus, we overcame the RMI limitations with this heuristic approach.

Counting the number of framed amino acids for each reduction strategy provides a simple metric of the results of these experiments. For the MI strategy, only nine AA always stay in the same group, while for the RMI strategy 19 of the 20 AA do. This simple metric shows how the objective function is more stable and thus the obtained alphabets are easier to understand, (for instance, most of the charged residues belong to the same reduction group). Moreover, later in this subsection we will show how this strategy also gives better performance when learning from the reduced alphabets. For the DualRMI strategy, 17 AA of the target residue and 15 of the other residues in the window remain in the same group. These numbers are less than the 19 AA of the RMI strategy, but RMI had no five-letter alphabet and therefore it is easier for this strategy to maintain the groups.

When optimizing for a two-letter alphabet, both MI and RMI find two identical groups of AA types that separate the hydrophobic residues (*ACFGHILMVWY*) from the rest (Histidine is sometimes considered as Hydrophobic [44]). Hydrophobicity is one of the main factors in the folding process of proteins, so it is natural that a reduction process into only two symbols is equivalent to identifying the hydrophobic residues. The DualRMI strategy also gives similar reduction groups, but with small differences between the reduction of the target residue, where the hydrophobic group is smaller, and the reduction for the other residues, where the hydrophobic group contains more AA types.

Looking at the physico-chemical properties of the four and five-letter alphabets a clear difference emerges (reflected in performance, as shown later) between the reduction groups for the MI strategy and those for the other two strategies. All MI groups have very mixed properties, while in RMI and DualRMI there is a clear difference between groups that include hydrophobic residues or not. However, some groups are difficult to explain, such as the *GHTS *group for the DualRMI five-letter alphabet. G, T and S are small amino acids, H is large. G and H are hydrophobic, while the other two are not. H is aromatic and has a high coil propensity.

A retrospective analysis of the dataset reveals why GHTS are clustered together. Table 3 shows, for each AA type, the proportion of residues in the dataset that belong to the high CN class, sorted by increasing order. The groups correlate almost perfectly with the sorted residues in the table, showing that G, H, T and S have similar properties in terms of CN.

**Table 3:** AA types sorted by the ratio of high CN residues in the dataset.

Amino Acid	High CN ratio	Red. group
K	7.0%	1
E	9.8%	1
D	13.4%	1
Q	14.9%	1
R	15.1%	1
N	18.6%	1
P	20.6%	1
S	25.3%	2
T	26.3%	2
H	27.6%	2
G	30.2%	2

Y	38.0%	3
W	40.8%	3
		
A	41.1%	4
M	43.4%	4
		
L	44.8%	3

F	45.8%	5
V	49.2%	5
I	50.9%	5
C	53.5%	5

Table also shows the reduction groups to which they belong for the DualRMI five-letter strategy.

The reduced alphabets were used to represent the CN datasets, which were then used to train and test BioHEL. Table 4 contains the results of the learning process. For each reduction strategy and alphabet size we have reported three measures. As a performance measure we have included the test accuracy. To illustrate the interpretability and explanatory power of the reduced datasets, we have included two complexity metrics for the classifiers: average number of rules and average number of relevant attributes in each rule. As a baseline, the performance and complexity obtained with the full AA type representation is also included (labelled orig.). These results were analyzed using statistical t-tests (99% conf.) to determine if the differences in accuracy were significant. The Bonferroni correction for multiple comparisons was employed.

**Table 4:** Performance of BioHEL in the CN datasets.

Strategy	Alphabet Size	% Accuracy	#Rules	#expr. atts.
Orig	20+1	74.0 ± 0.6	34.4 ± 1.7	9.0 ± 0.1

MI	2	72.3 ± 0.6•	21.4 ± 1.0	6.2 ± 0.7
	3	73.2 ± 0.6	30.2 ± 1.7	6.7 ± 1.0
	4	72.4 ± 0.8•	26.4 ± 2.1	7.1 ± 1.1
	5	71.8 ± 0.9•	23.4 ± 4.8	7.8 ± 1.0

RMI	2	72.3 ± 0.6•	21.4 ± 1.0	6.2 ± 0.7
	3	73.2 ± 0.6	30.2 ± 1.7	6.7 ± 1.0
	4	73.3 ± 0.5	30.2 ± 1.5	6.1 ± 1.1
	5	--	--	--

DualRMI	2	72.4 ± 0.5•	24.0 ± 1.3	7.0 ± 1.0
	3	73.0 ± 0.6•	29.1 ± 1.6	6.5 ± 1.1
	4	73.3 ± 0.6	29.7 ± 1.3	6.3 ± 1.0
	5	73.3 ± 0.5	30.4 ± 1.1	6.2 ± 1.1

Accuracy is the average test accuracy from the ten cross-validation folds. A • marks reduced datasets where performance is significantly worse than the original full AA representation according to statistical t-tests with 99% confidence level.

The results for the MI strategy are similar to those presented in previous work. Only the dataset with the three-letter alphabet gives a performance statistically comparable to the performance given by the original dataset. Larger alphabet sizes degraded the results. On the other hand, both RMI and DualRMI obtain their best results in the four and five-letter alphabets, closing the performance gap with the full AA alphabet to 0.7% (for the DualRMI strategy and four-letter alphabet). DualRMI always obtains better results than RMI, except for the three-letter case, showing the usefulness of this strategy. These results (together with the results for the RSA dataset, shown later in this section) answer the first of the questions that we wanted to address in this paper, determining the minimum alphabet size that obtains similar results to the full AA representation. Moreover, the rules learned using the reduced datasets are more compact and human readable, in both the number of rules and the number of expressed attributes, than the solutions produced from the original dataset.

### Relative Solvent Accessibility prediction

**Figure 2:**
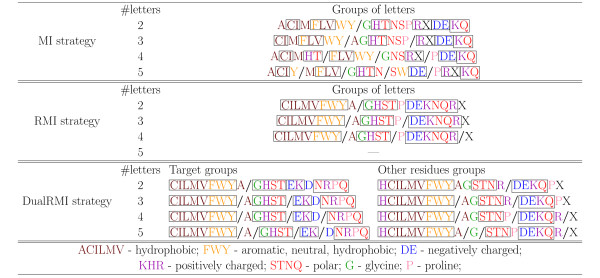
**Alphabet reductions for the Solvent Accessibility feature**. Groups are separated by '/'. Solid rectangle marks amino acids that remain in the same group for all four alphabets.

ECGA, with each of the three reduction strategies (MI, RMI and DualRMI) was also used to find alphabet reductions to two, three, four and five symbols for RSA prediction. Figure 2 describes the reductions obtained, using the same visualization techniques employed previously. The comparison between the alphabets in figure 1 with those in figure 2, generated for CN, shows several differences. For instance, the alphabets for RSA contains more groups of polar residues, while most of the hydrophic residues are grouped together. On the other hand, several groups of hydrophobic residues exist in the CN alphabets, while the polar residues are grouped together.

Given that both features are strongly anti-correlated (Pearson's correlation coefficient of -0.55 for our dataset), one could expect that the reduced alphabets found for CN would be useful for RSA too. This turns out not to be the case and the differences between the two features are successfully captured by the alphabet reduction process. Moreover, these differences further justify the automated procedure presented in this paper, which tailors the reduction specifically to each problem.

The number of amino acids that remain grouped together in the MI strategy is 13, while in the RMI strategy it is 18. For the DualRMI strategy, 18 AA types also stay in the same group for the target residue, while 16 of them do for the other residues. Again, no alphabet with five letters was found for the RMI strategy and we find, again, a group of AA types formed by *GHTS *in RMI and DualRMI alphabets. If we sort the amino acids by their proportion of exposed instances in the dataset (reported in table 5) we can see how the reduction groups match perfectly the order of AA types in the table, similarly to what happened in the case of the CN dataset.

**Table 5:** AA types sorted by the ratio of exposed residues in the RSA dataset.

Amino Acid	Exposed ratio	Red. group
C	12.8%	1
I	15.4%	1
F	15.9%	1
L	19.5%	1
V	20.3%	1
W	21.3%	1
M	23.2%	1
Y	25.6%	1

A	33.6%	2

G	43.5%	3
H	43.8%	3
T	47.1%	3
S	48.0%	3

P	55.1%	4
N	58.3%	4
R	61.3%	4
Q	62.5%	4
D	64.7%	4

E	73.1%	5
K	81.9%	5

Also showing the reduction group to which each AA type belongs for the DualRMI strategy and five-letter alphabet.

We also observe some interesting facts that are particular to this dataset, namely the presence of reduction groups including only one amino acid type: the *A *type for the DualRMI strategy with five-letter alphabet and target residue and G for the other residues, and X in RMI with four letters and DualRMI with four and five letters. The end-of-chain symbol, X, only appears in 3.2% of the instances in the dataset. However, this symbol has the highest proportion, 72.4%, of exposed instances of all 21 alphabet letters. In comparison, the second letter with highest proportion of exposed residues is Lys with only 49.9% of exposed instances. Thus, it makes sense for the protocol to create a reduction group containing only X. We also observe other very small groups, like *EK*.

The reduced alphabets were used to represent the RSA datasets, which were then used to train and test BioHEL. Table 6 contains the results of the learning process, which are quite different from the results for CN. Here only the DualRMI strategy (using the four and five-letter alphabets) achieves a performance statistically similar to that obtained by learning the dataset with full alphabet. There is a significant performance difference between RMI and DualRMI in the RSA, which was not observed for the CN dataset. Moreover, the performance gap between the best reduced alphabet, using DualRMI and five letters and the original alphabet with full AA type representation is 0.4%, even less than for the CN dataset. For the predictions of RSA, an alphabet representation with only two or three symbols degrades the classification performance severely. This matches with our previous observation of having very small reduction groups in some of the DualRMI alphabets (such as *A*, *G *or *EK*). If an alphabet of two or three letters contains one letter with such small groups it means that there must be another letter grouping a large number of letters, and the likelihood that such large group is meaningful is very low, because it is over-simplifying the representation. Table 7 shows two rule sets generated by BioHEL, one for CN, the other for RSA, both obtained from the full AA alphabet. We can observe very specific predicates associated to the target residue, especially for the RSA dataset. It would be practically impossible to generate equivalent rules when learning from alphabets with only two or three letters.

**Table 6:** Performance of BioHEL in the RSA datasets.

Strategy	Alphabet Size	% Accuracy	#Rules	#expr. atts.
Orig.	20+1	70.7 ± 0.4	58.6 ± 2.3	9.0 ± 0.2

MI	2	67.6 ± 0.3•	52.9 ± 4.2	5.8 ± 1.3
	3	69.4 ± 0.3•	54.9 ± 1.1	5.4 ± 1.2
	4	68.9 ± 0.6•	54.5 ± 1.3	5.9 ± 1.2
	5	67.9 ± 0.9•	53.1 ± 3.8	6.8 ± 1.2

RMI	2	67.6 ± 0.3•	52.9 ± 4.2	5.8 ± 1.3
	3	69.7 ± 0.4•	56.5 ± 1.3	5.5 ± 1.2
	4	69.9 ± 0.4•	57.5 ± 1.2	6.3 ± 1.4
	5	--	--	--

DualRMI	2	66.6 ± 0.4•	33.4 ± 4.8	3.7 ± 0.8
	3	69.9 ± 0.4•	56.7 ± 1.3	5.3 ± 1.1
	4	70.1 ± 0.4	58.0 ± 1.2	6.0 ± 1.4
	5	70.3 ± 0.4	58.2 ± 1.1	6.5 ± 1.6

Accuracy is the average test accuracy from the ten cross-validation folds. A • marks reduced datasets where performance is significantly worse than the original full AA representation, according to the statistical t-tests with a 99% confidence level.

**Table 7:** Rule-sets obtained by BioHEL for CN and RSA predictions using the full AA alphabet.

**Rules for CN prediction**	**Rules for RSA prediction**
1:If *AA*_-4 _∉ {E,L,M,N,R,X}, *AA*_-3 _∉	1:If *AA*_-4 _∉ {G,I,L,V,X,F,Y}, *AA*_-3 _∉
{D,E,N,H,R,F,W,Y,X}, *AA*_-2 _∉ {E,F,W,N,S,P},	{G,Q,F,W}, *AA*_-2 _∉ {C,N,P}, *AA*_-1 _∉ {A,I,V,Q,Y}, *AA *∈ {K},
*AA*_-1 _∉ {D,E,F,G,H,K,N,Q}, *AA *∉ {C,I,L,M,V}, *AA*_1 _∉	*AA*_1 _∉ {F,I,L,M,V,N,T,P}, *AA*_2 _∉ {N,Q,S,P}, *AA*_3 _∉
{D,E,K,R,N,Q,S,P}, *AA*_2 _∉ {H,R,M,P,T,N,W,X}, *AA*_3 _∉	{C,I,L,R,W}, *AA*_4 _∉ {A,C,I,L,R,S} then RSA is high
{A,C,I,L,M,V,F,G,H,X}, *AA*_4 _∉ {A,C,L,M,G,H,F,W} then	2:If *AA*_-4 _∉ {A,I,L,V,G,W,F}, *AA*_-3 _∉
CN is High	{C,I,M,V,G,P,S,T,Y,F}, *AA*_-2 _∉ {C,H,R,F,W}, *AA*_-1 _∉
2:If *AA*_-4 _∉ {E,H,K,R,N,Q,P,W,X}, *AA*_-3 _∉	{F,H,I}, *AA *∈ {E,K}, *AA*_1 _∉ {I,M,V,N,S}, *AA*_2 _∉
{D,E,K,R,M,N,T,P,Y}, *AA*_-2 _∉ {D,N,S}, *AA*_-1 _∉	{C,D,H,N,S}, *AA*_3 _∉ {A,C,I,L,V,H,N,W,Y,F}, *AA*_4 _∉
{D,E,G,K,N,P}, *AA *∈ {A,C,I,L,M,W}, *AA*_1 _∉	{G,H,I,L,M,P,F,W,Y} then RSA is high
{D,E,G,K,P,N,Q,S,T}, *AA*_2 _∉ {C,I,D,G,P,S,X,Y}, *AA*_3 _∉	.
{D,E,G,K,R,N,Q,S,P,X}, *AA*_4 _∈ {A,C,I,L,M,V,F,G,T} then	.
CN is high	.
.	.
.	.
.	.
32:If *AA*_-4 _∉ {E,F,P,K,R,S,X}, *AA*_-3 _∈	.
{A,C,I,L,V,G,F,W,X,Y}, *AA*_-2 _∉ {F,H,I,M,P,N,Q,X}	57:If *AA*_-4 _∉ {Q}, *AA*_-3 _∉ {G,H,I,V,P,Y}, *AA*_-2 _∉
*AA*_-1 _∉ {C,I,D,E,G,P,K,R,N,S}, *AA *∈	{G,H,T,M,V,W,Y,F}, *AA*_-1 _∉ {G,I,M,V,X,Y},
{A,C,I,L,M,V,F,W,Y}, *AA*_1 _∉ {G,P,N,Q,T,X}, *AA*_2 _∉	*AA *∈ {D,E,G,H,K,P,Q,S}, *AA*_1 _∉ {E,F,W}, *AA*_2 _∈
{D,G,N,Q,S}, *AA*_3 _∉ {D,K,P,Q,W}, *AA*_4 _∉ {A,I,M,R,X}	{D,E,G,H,K,N,S,T,P,X}, *AA*_3 _∉ {G,K,F,W}, *AA*_4 _∉
then CN is high	{L,M,R,W} then RSA is high
33:Default class: CN is low	58:Default class: RSA is low

Rule set at the left is for CN prediction. Rule set at the right is for RSA prediction. *AA*_± *n *_means AA type for residue in position ± *n *in respect to the target residue. X means end of chain, in case one of the residues of the window overlaps with either one of the two ends of a chain.

### Comparison against other reduced alphabets

We compared the performance of the five-letter reduced alphabets obtained using the DualRMI strategy, the most successful of those presented in this paper, against other alphabets summarized in table 8. The first four are taken from the literature [16]. For the specific usage in this work it has been necessary to extend them with an extra letter, the end-of-chain symbol (X), which is used to identify when the window of residues around the target overlaps with one end of the chain. We would like to clarify that these alphabets from the literature were not optimized for CN or RSA prediction, but for other tasks such as sequence alignment. Thus, we expect our alphabets to perform better because they have been explicitly optimized for the features at hand. Our goal in this experiment is to check how adaptable these reduced alphabets are across different PSP-related problems. Our aim is, by no means, to find a single best alphabet that is good for all protein problems.

**Table 8:** Reduced alphabets compared to the DualRMI strategy.

Name	Description	#letters	Reference
WW5	AHT/CFILMVWY/DE/GP/KNQRS/X	6	[9]
SR5	AEHKQRST/CFILMVWY/DN/G/P/X	6	[6]
MU4	AGPST/CILMV/DEHKNQR/FYW/X	5	[46]
MM5	AG/C/DEKNPQRST/FILMVWY/H/X	6	[16]
HD1	AV/CGNP/D/EKRQ/FWYH/ILM/ST/X	8	This work
HD2	AV/CGNP/D/EKR/Q/FWY/H/ILM/ST/X	10	This work
HD3	AV/C/GNP/D/EKR/Q/FWY/H/IL/M/ST/X	12	This work

These alphabets have been extended with a letter corresponding to the end of chain symbol. HD *n *= Human Designed alphabet *n*.

Moreover, we have manually designed three reduced alphabets based on physico-chemical properties. The first alphabet uses four properties: size, hydrophobicity, coil propensity and aromatic nature. The second alphabet adds another property to the first one: charged residues, and the third alphabet adds another property to the second one: those amino acids having sulphur atoms. The goal of this comparison is to check how far in performance terms a *generic *reduced alphabet is from an optimized reduced alphabet. Table 9 contains these comparisons. The automatically derived reduced alphabets obtain higher performance than all the other reduced alphabets. The performance degradation of these alternative alphabets, when compared to the full AA representation, was significant according to the t-tests for all the alphabets in the RSA dataset, and for the MU4 alphabet in the CN dataset. The three human-designed alphabets, despite having more letters than the other alphabets, perform poorly. A less reduced alphabet, if improperly designed, will lose as much information or more than a more compact alphabet.

**Table 9:** Performance of BioHEL for the compared reduced alphabets in the CN and RSA datasets.

Alphabet	% Acc. on CN dataset	% Acc. on RSA dataset
AA	74.0 ± 0.6	70.7 ± 0.4
DualRMI	73.3 ± 0.5	70.3 ± 0.4
WW5	73.1 ± 0.7	69.6 ± 0.4•
SR5	73.1 ± 0.7	69.6 ± 0.4•
MU4	72.6 ± 0.7•	69.4 ± 0.4•▼
MM5	73.1 ± 0.6	69.3 ± 0.3•▼
HD1	72.9 ± 0.6	69.3 ± 0.4•▼
HD2	73.0 ± 0.6	69.3 ± 0.4•▼
HD3	73.2 ± 0.6	69.9 ± 0.4•

A • marks reduced datasets where BioHEL performs significantly worse than the AA type dataset according to the t-tests with a 99% confidence level. A ▼ marks the alphabets that performed significantly worse than the DualRMI strategy.

Even though that the performance differences between the other reduced alphabets and the AA representation were significant, these differences are not very large. This observation might be taken to suggest that developing new reduced alphabets adapted for CN or RSA may not be worthiwhile. We do not agree with this contention for several reasons. First of all, the automated alphabet reduction proposed in this paper is very generic and flexible. Thus, generating new reduced alphabets tailored to new features can be done easily and without much effort. Secondly, the previous alphabets, despite still being able to hold quite a large amount of information, are not able to capture totally the crucial information for an optimal prediction of CN and RSA. To illustrate this claim we have regenerated tables 3 and 5 (that showed the reduction groups of the DualRMI strategy sorted by the ratio of high CN/exposed residues in the dataset) to compare our DualRMI alphabets to the four alphabets from the literature. The new tables are table 10 (for CN) and table 11 (for RSA).

**Table 10:** Comparison of reduced alphabets in terms of the ratio of high CN in the dataset by AA type.

Amino Acid	High CN ratio	DualRMI	WW5	SR5	MU4	MM5
K	7.0%	1	1	1	1	1
E	9.8%	1	2	1	1	1
D	13.4%	1	2	2	1	1
Q	14.9%	1	1	1	1	1
R	15.1%	1	1	1	1	1
N	18.6%	1	1	2	1	1
P	20.6%	1	3	3	2	1
S	25.3%	2	1	1	2	1
T	26.3%	2	4	1	2	1
H	27.6%	2	4	1	1	2
G	30.2%	2	3	4	2	3
Y	38.0%	3	5	5	3	4
W	40.8%	3	5	5	3	4
A	41.1%	4	4	1	2	3
M	43.4%	4	5	5	4	4
L	44.8%	3	5	5	4	4
F	45.8%	5	5	5	3	4
V	49.2%	5	5	5	4	4
I	50.9%	5	5	5	4	4
C	53.5%	5	5	5	4	5

Trans.	--	5	9	9	8	6

Ave. range	--	8.7%	14.0%	12.6%	16.8%	10.2%

Trans. = number of transitions between groups. Ave. range = average range of each reduction group, range is the difference between the maximum and minimum High CN ratio of the AAs of a group.

**Table 11:** Comparison of reduced alphabets in terms of the ratio of exposed residues in the dataset by AA type Trans. = number of transitions between groups.

Amino Acid	Exposed ratio	DualRMI	WW5	SR5	MU4	MM5
C	12.8%	1	1	1	1	1
I	15.4%	1	1	1	1	2
F	15.9%	1	1	1	2	2
L	19.5%	1	1	1	1	2
V	20.3%	1	1	1	1	2
W	21.3%	1	1	1	2	2
M	23.2%	1	1	1	1	2
Y	25.6%	1	1	1	2	2
A	33.6%	2	2	2	3	3
G	43.5%	3	3	3	3	3
H	43.8%	3	2	2	4	4
T	47.1%	3	2	2	3	5
S	48.0%	3	4	2	3	5
P	55.1%	4	3	4	3	5
N	58.3%	4	4	5	4	5
R	61.3%	4	4	2	4	5
Q	62.5%	4	4	2	4	5
D	64.7%	4	5	5	4	5
E	73.1%	5	5	2	4	5
K	81.9%	5	4	2	4	5

Trans.	--	4	8	8	9	4

Ave. range	--	7.0%	16.0%	9.4%	19.9%	11.0%

Ave. range = average range of each reduction group, range is the difference between the maximum and minimum exposed ratio of the AAs of a group.7

To quantify the differences between the reduced alphabets we have computed two simple statistics: (1) counting the number of transitions between reduction groups through the sorted list of AA and (2) computing the range of each reduction group, that is, the difference between the maximum and minimum high CN/exposed ratio of the AAs in the same group, and averaging these ranges. Both a low number of transitions and a small average range indicate that the AA grouped together have similar behavior in terms of CN or RSA. The alphabets generated by our DualRMI strategy present always the lowest number of transitions and average range from all the compared alphabets. Except for the MM5 alphabet, all the other three alphabets from the literature have a number of transitions that, at least, almost doubles the number of groups of DualRMI.

With these statistics we have quantified the differences of the compared alphabets in relation to CN and RSA. However, why do several of these alphabets have poor performance for CN or RSA? Tables 10 and 11 can also answer this question. In MM5, Lys (with 81.9% of exposed residues) and Thr (with 47.1% of exposed residues) are in the same group. For MU4, Lys and His (43.8% of exposed residues) are in the same group. For SR5, Lys is in the same group as Ala (33.6% of exposed residues. Finally, for WW5 Lys is in the same group as Ser (48.0% of exposed residues). These ranges of exposed ratios are very large, effectively treating equally AA types with very different behavior. In DualRMI this situation does not happen, and the largest range is only 12.8% wide. For the CN dataset all ranges of high CN ratio for all alphabets are much smaller, and this is reflected in the much smaller performance differences between all reduced alphabets. The principal difference between the alphabet with worse performance (MM4) and the others, besides having one less letter, is having clustered together Ala and Pro. None of the other alphabets has both AAs in the same group. A further investigation would be necessary to quantify the impact of such a group.

As a final experiment, we test the performance of the reduced alphabet obtained from the RSA dataset in learning CN and also using the CN reduced alphabet to learn RSA. This latter option is labelled *DualRMI-alt*. The results of this experiment are in table 12. The optimized alphabet using DualRMI for RSA performed well when applied to the CN feature. However, the reverse is not the case. The alphabet optimized for CN data performs poorly when applied to the RSA data. The reason for this is that the CN alphabet misses a specific reduction group only for Glu and Lys and, in consequence, having the same problem that we have discussed above about the alphabets from the literature. The rule sets in table 7 also showed this difference between CN and RSA. The predicates associated to the target residue were quite different between features. This observation shows that reduced alphabets should be *tailored *to the specific problem at hand, even if the problems are as similar as CN and RSA.

**Table 12:** Performance of BioHEL on learning CN and RSA using the alphabet optimized for the other dataset.

Alphabet	% Acc. on CN dataset	% Acc. on RSA dataset
AA	74.0 ± 0.6	70.7 ± 0.4
DualRMI	73.3 ± 0.5	70.3 ± 0.4
DualRMI-alt	73.3 ± 0.7	69.53 ± 0.7•

A • marks reduced datasets where BioHEL obtains a performance significantly worse than the AA type dataset according to the statistical t-tests with a 99% confidence level. No significant differences were detected between the reduced alphabets.

### Extrapolating the obtained alphabets to reduce an evolutionary information-based representation

The experiments performed so far have been applied to proteins represented using their primary sequence. This representation contains limited amount of information when compared to other, more modern, approaches such as PSSM representations that take into account evolutionary information of proteins. In the final experiments reported in this paper we now evaluate the generality of the results (reduced alphabets) obtained so far by our protocol. To do so we are going to adapt the alphabet reduction process from the primary sequence representation to a PSSM representation.

**Figure 3:**
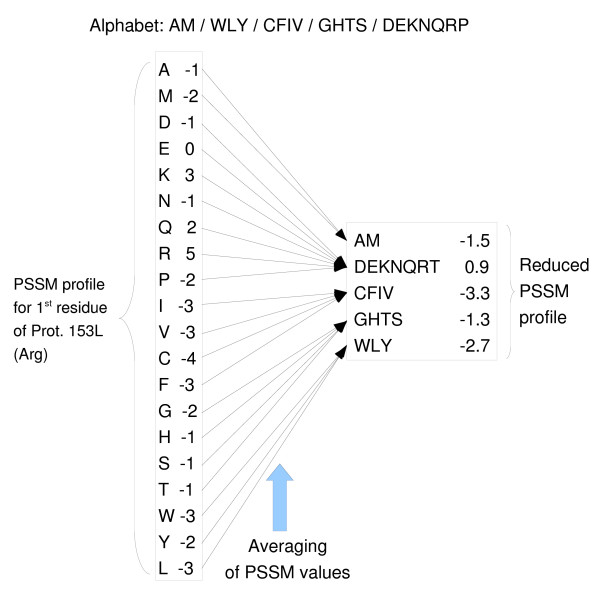
**Alphabet Reduction process adapted to the Position-Specific Scoring Matrix residue representation**.

In the primary sequence representation, each residue is characterized by a single discrete variable with 20 possible values (the AA alphabet). On the other hand, in the PSSM representation, each residue is characterized by 20 continuous variables. Each of the 20 variables indicates, for the associated AA type, its degree of preference for the corresponding residue position in the chain. Each letter in a reduced alphabet groups a set of AA types that should be treated as if they all were the same. Given all this, the application of our current reduced alphabet to a PSSM representation (represented in figure 3) is very simple:

• Each residue will be characterized by *N *continuous variables, where *N *is the number of letters of our reduced alphabet.

• The value of each of these attributes will be computed by averaging the variables of the PSSM profile associated to the AA types that were grouped to form that letter of the reduced alphabet.

• In the case of using a dual alphabet (from the DualRMI strategy), we will apply one reduction to the PSSM profile of the target residue and the other reduction to the PSSM profiles of the other residues in the window.

As an example, if we were to apply this process, using the five-letter DualRMI alphabet, we would take the original dataset of 180 variables (20 PSSM values × 9 window positions) and transform it into a dataset of 37 variables, that is 5 variables for the target residue and 4 variables for the other 8 window positions. As we explained previously, the RMI and DualRMI (for the non-target alphabet) strategies converged to four-letter alphabets even if they were trying to optimize a five-letter alphabet.

The validation of this reduced PSSM representation is the same as used before. We will train BioHEL to predict CN and RSA using two representations: (1) the original PSSM representation of 180 attributes and (2) the reduced PSSM representation (using the DualRMI strategy) consisting of 37 attributes and we will compare the obtained accuracies. Table 13 contains the results of these experiments. We show four different performance metrics. Besides reporting test accuracy, and number of rules and number of expressed attributes per rule, as we did in the previous experiments, we also report the average run-time of each BioHEL training process. A full experiment consists of 400 runs of BioHEL: 10 cross-validation training sets × 40 models in each ensemble. The reported run-time metric is the average of these 400 runs. This metric is relevant in this context, because the training process using the reduced representation is much lower, and given the magnitude of the time values, this run-time reduction becomes an important factor. All experiments were performed on the Jupiter supercomputer of the University of Nottingham, using Opteron-248 processors running at 2.2 GHz, the Linux operating system and a C++ implementation of BioHEL.

**Table 13:** Performance of BioHEL on learning CN and RSA using the reduced PSSM representation

Dataset	Representation	Accuracy	#Rules	#expr. atts.	Run-time(h)
CN	PSSM	81.4 ± 0.4	269.5 ± 12.7	14.4 ± 3.1	31.4 ± 2.9
	Reduced PSSM	80.8 ± 0.3	256.0 ± 12.1	12.9 ± 2.7	19.4 ± 1.7

SA	PSSM	78.5 ± 0.4	520.4 ± 15.3	13.9 ± 3.5	54.3 ± 4.2
	Reduced PSSM	77.6 ± 0.4	493.5 ± 14.9	12.9 ± 3.3	34.3 ± 3.0

The results reported in table 13 indicate that the performance gap between both representations, like in the previous experiments is small: 0.6% for CN, 0.9% for RSA. Moreover, the solutions generated by BioHEL when learning from the reduced representation are more compact in terms of number of rules and number of variables used in each rule. The number of variables per rule for these experiments is only a fraction (very small for the PSSM representation) of the attributes. Moreover, the run-time of the training process was approximately 1.6 times faster for both datasets, and considering that the run-times are reported in hours, this reduction is quite considerable in absolute terms.

The predictability of these models is quite comparable to other work from the literature. Kinjo et al [30] report 76.3% accuracy on two-state CN prediction, although their class definition criterion is slightly different from ours, and they use a distance threshold of 12 Å instead of 10 Å. Dor and Zhou [45] recently obtained 78.6% accuracy on two-state RSA prediction from a PSSM representation (accuracy was increased to 79.2% with additional input information) with a 25% RSA cutoff, the same as we have employed in this paper. Thus, our alphabet reduction protocol can be applied to state-of-the-art representations and obtains accuracy levels that are quite similar to the performance reported in the literature for predicting CN and RSA. This last experiment has helped to illustrate the generality of our findings.

## Conclusion

This paper develops the use of information theory based automated procedures for alphabet reduction in PSP datasets. Our investigations indicate that: (1) finding a reduced alphabet with a performance that is statistically equivalent to the performance obtained with the full AA type representation is possible, (2) this does not compromise accuracy and enhances interpretability and (3) different problems might require different reductions and (4) the alphabets obtained from primary sequence data can be successfully adapted to richer representations using evolutionary information. These four observations taken together point to the need for a robust automated method, such as the one described in this paper, for tackling alphabet reduction. The tests with the three reduction strategies tell us that the DualRMI strategy can give a significant advantage, as has been shown for the solvent accessibility dataset. The reduction groups that our automated procedure finds translate quite well to physico-chemical characteristics such as hydrophobicity, but not completely. We find groups of letters such as GHTS that are difficult to explain. A retrospective analysis of the dataset shows that the reduction groups are sound, because they are a consequence of the underlying data that we are mining. Moreover, the learned reductions suggested a new way of interpreting the data, which a priori would have made no sense. That is, our results show that this automated procedure is sound, is not bound by any preconceptions the experimenter might have, tailors the alphabet reduction specifically for each dataset and obtains higher performance than other reduced alphabets available in the literature or human-design alphabet reductions. It can be applied with performance comparable to state-of-the-art protein representations, while at the same time it is able to provide new insight into the available data.

In future work, we will test alternative objective functions as well as other robust MI estimations. It would also be interesting to check if it is possible to find alphabets of slightly higher cardinality than the ones studied in this paper that are able to close even more the performance gap with the original representation. We also would like to apply this protocol to protein design problems. Finally, it would be interesting to apply this protocol to datasets with more than two classes or, even, regression datasets, although we expect that this option will require a much more robust fitness function, as this means (a) making worse the sample size problem of the MI metric and (2) MI has to be estimated in some way if applied to regression problems, as it originally only deals with discrete variables.

## Authors' contributions

JB participated in the conception and design of the study, carried out the experiments and statistical analysis reported in this paper and drafted the manuscript. MS helped to draft the manuscript. JH participated in the design of the study and helped to draft the manuscript. RS and AV participated in the design of the study. NK participated in the conception and design of the study and helped to draft the manuscript. All authors read and approved the final manuscript.

## Supplementary Material

Additional file 1 **Reduction groups obtained for all the training sets**. This document lists 6 tables (3 reduction strategies and two datasets) containing the details of the reduction groups generated by our protocol for each of the ten training sets. Click here for file
