# Supplementary material for: Automated Alphabet Reduction for Protein Datasets
Source: BMC Bioinformatics. 2009 Jan 6;10:6. doi: 10.1186/1471-2105-10-6 (PMC2646702; doi:10.1186/1471-2105-10-6)
Supplement: Additional file 1 — Reduction groups obtained for all the training sets. This document lists 6 tables (3 reduction strategies and two datasets) containing the details of the reduction groups generated by our protocol for each of the ten training sets. [file 1471-2105-10-6-S1.pdf]

Automated Alphabet Reduction for Protein Datasets Supplementary Material

Jaume Bacardit*[1,2], Michael Stout[1,2], Jonathan D. Hirst[3], Alfonso Valencia[4], Robert E. Smith[5] and Natalio Krasnogor*[1]

[1]ASAP research group, School of Computer Science, University of Nottingham, Jubilee Campus, Wollaton Road, Nottingham, NG8 1BB, UK
[2]MYCIB, School of Biosciences, University of Nottingham, Sutton Bonington, LE12 5RD, UK
[3]School of Chemistry, University of Nottingham, University Park, Nottingham, NG7 2RD, UK
[4]Spanish National Cancer Research Centre, Melchor Fdez Almagro, 3. 28029 Madrid, Spain
[5]Dept. of Computer Science, University College London, Gower Street, London, WC1E 6BT, UK

Email: JB*- jaume.bacardit@nottingham.ac.uk; MS - mqs@cs.nott.ac.uk; JDH - jonathan.hirst@nottingham.ac.uk; AV - valencia@cnio.es; RES - robert.elliott.smith@gmail.com; NK*- natalio.krasnogor@nottingham.ac.uk;

*Corresponding author

Table 1: Alphabet reductions for all training partitions and MI Strategy for the CN dataset

Alphabet size	Training set	Groups of letters
2	1-10	ACFGHILMVWY/DEKNPQRSTX
3	1	ACFILMVY/DEKNPQRX/GHSTW
	2-10	ACFILMVWY/DEKNPQRX/GHST
4	1	ACIMV/DEKPRX/FHLNWY/GQST
	2,3	AFHTY/CILMV/DEKPQX/GNRSW
	4,9	AFMSY/CHILV/DEKPQX/GNRTW
	5	AFVY/CILMSW/DEKQRX/GHNPT
	6	AFMSY/CILVW/DGHNR/EKPQTX
	7	AFHNWY/CILMV/DEKQRX/GPST
	8,10	AFTWY/CILMV/DEKPQX/GHNRS
5	1	ACIR/DENQX/FLMS/GKPW/HTVY
	2	ACFI/DKPQX/EHRSW/GMNT/LVY
	3	ACNP/DEKQX/FLTW/GHRS/IMVY
	4	AFT/CGHIM/DEKQX/LRSW/NPVY
	5	AIT/CLVW/DKQRX/ENPS/FGHMY
	6	AFV/CHLSW/DEKQX/GIMY/NPRT
	7	AFMWY/CHLT/DNPR/EKQSX/GIV
	8	AHVW/CFLS/DEKQX/GIMY/NPRT
	9	ACMV/DENQX/FLTW/GHIY/KPRS
	10	ACMV/DEPQX/FLTW/GHIY/KNRS

Table 2: Alphabet reductions for all training partitions and RMI Strategy for the CN dataset

Alphabet size	Training set	Groups of letters
2	1-10	ACFGHILMVWY/DEKNPQRSTX
3	1-10	ACFILMVWY/DEKNPQRX/GHST
4	1-6,8-9	ACLM/DEKNPQRSTX/FIVW/GHY
	7,10	ALMY/CFIV/DEKNPQRSTX/GHW

Table 3: Alphabet reductions for all training partitions and DualRMI Strategy for the CN dataset

Alphabet size	Training set	Groups of letters
2	Target	
	1-10	ACFILMVWY/DEGHKNPQRSTX
	Other residues	
	1-10	ACFGHILMSTVWY/DEKNPQRX
3	Target	
	1-2,4-6,8-10	ACFILMVWY/DEKNQR/GHPSTX
	3,7	ACFILMVWY/DEKNPQR/GHSTX
	Other residues	
	1-2,5-6,9	ACGHSTW/DEKNPQRX/FILMVY
	3,7	AGHSTW/CFILMVY/DEKNPQRX
	4	AGHSTWY/CFILMV/DEKNPQRX
	8,10	ACGHSTWY/DEKNPQRX/FILMV
4	Target	
	1,7,10	AMW/CLY/DEGHKNPQRST/FIV
	2	ALM/CFI/DEGHKNPQRST/VWY
	3,5	AM/CFIVW/DEGHKNPQRST/LY
	4	AWY/CFILMV/DEKNPQR/GHST
	6	ALY/CFI/DEGHKNPQRST/MVW
	8	AW/CFILMVY/DEKNPQR/GHST
	9	AM/CFIV/DEGHKNPQRST/LWY
	Other residues	
	1,7,10	ACLY/DEKNPQRSX/FIMV/GHTW
	2-3,5-6	ACLM/DEKNPQRSX/FIVW/GHTY
	4	ACGHMTWY/DEKNPQRSX/FILV
	8	ACGHWY/DEKNPQRSTX/FILMV
	9	ACHLW/DEKNPQRSX/FIMV/GTY
5	Target	
	1,3	AM/CFIV/DEKNPQR/GHST/LWY
	2	AMW/CFI/DEHKNPQRST/GY/LV
	4,10	AMW/CFIV/DEKNPQR/GHST/LY
	5	AM/CIV/DEHKNPQRST/FLW/GY
	6	AY/CFMVW/DEKNPQR/GHST/IL
	7	AM/CFWY/DEKNPQR/GHST/ILV
	8	AMW/CFL/DEKNPQRST/GHY/IV
	9	AM/CFLW/DEKNPQRST/GHY/IV
	Other residues	
	1-2,4-5,9	ALMY/CGHW/DEKNPQRSTX/FIV
	3,6-8,10	ACLMY/DEKNPQRSTX/FIV/GHW

Table 4: Alphabet reductions for all training partitions and MI Strategy for the RSA dataset

Alphabet size	Training set	Groups of letters
2	1-10	ACFILMVWY/DEGHKNPQRSTX
3	1-10	AGHNPST/CFILMVWY/DEKQRX
4	1	ACHIMT/DEKPQ/FLVWY/GNRSX
	2	AHMVWY/CFILS/DEKQR/GNPTX
	3	AHMTWY/CFILV/DEKNQ/GPRSX
	4	AFHTY/CILMVW/DEKPQ/GNRSX
	5	AFHSY/CILMVW/DGNT/EKPQRX
	6	AFGMW/CILVY/DEKNQ/HPRSTX
	7	AGMWY/CFILV/DEKNQ/HPRSTX
	8	AGMWY/CFILV/DEKQRX/HNPST
	9	AIVY/CFHLMSW/DGNT/EKPQRX
	10	AFGY/CILMVW/DHNST/EKPQRX
5	1	ACIR/DENQX/FLMS/GKPW/HTVY
	2	ACFI/DKPQX/EHRSW/GMNT/LVY
	3	ASAP/DEKQX/FLTW/GHRS/IMVY
	4	AFT/CGHIM/DEKQX/LRSW/NPVY
	5	AIT/CLVW/DKQRX/ENPS/FGHMY
	6	AFV/CHLSW/DEKQX/GIMY/NPRT
	7	AFMWY/CHLT/DNPR/EKQSX/GIV
	8	AHVW/CFLS/DEKQX/GIMY/NPRT
	9	ACMV/DENQX/FLTW/GHIY/KPRS
	10	ACMV/DEPQX/FLTW/GHIY/KNRS

Table 5: Alphabet reductions for all training partitions and RMI Strategy for the SA dataset

Alphabet size	Training set	Groups of letters
2	1-10	ACFILMVWY/DEGHKNPQRSTX
3	1,3-4,8-9	AGHPST/CFILMVWY/DEKNQRX
	2,6	AGHNPST/CFILMVWY/DEKQRX
	5,7,10	AGHST/CFILMVWY/DEKNPQRX
4	1-10	AGHST/CFILMVWY/DEKNPQR/X

Table 6: Alphabet reductions for all training partitions and DualRMI Strategy for the SA dataset

Alphabet size	Training set	Groups of letters
2	Target	
	1-10	ACFILMVWY/DEGHKNPQRSTX
	Other residues	
	1,3-10	ACFGHILMNRSTVWY/DEKPQX
	2	ACFGHILMTVWY/DEKNPQRSX
3	Target	
	1-10	AGHST/CFILMVWY/DEKNPQR
	Other residues	
	1-9	AGNRST/CFHILMVWY/DEKPQX
	10	AGHNRST/CFILMVWY/DEKPQX
4	Target	
	1	AGHST/CFILMVWY/DEK/NPQR
	2-3,6-8,10	AY/CFILMVW/DEKNQR/GHPST
	4	AY/CFILMVW/DEKQR/GHNPST
	5,9	AH/CFILMVWY/DEKNQR/GPST
	Other residues	
	1-2	AGNPST/CFHILMVWY/DEKQR/X
	3,5-7,9	AGNST/CFHILMVWY/DEKPQR/X
	4	AGRST/CFHILMVWY/DEKNPQ/X
	8	AGPST/CFHILMVWY/DEKNQR/X
	10	ACFILMVWY/DEKPQ/GHNRST/X
5	Target	
	1,5-8	A/CFILMVWY/DNPQR/EK/GHST
	2-3,6,9	AY/CFILMVW/DEK/GHST/NPQR
	4	AY/CFILMVW/DEKQ/GHST/NPR
	10	AY/CFILMVW/DNPQR/EK/GHST
	Other residues	
	1,5,7-8	ACFHILMVWY/DEKNPQRST/G/X
	2,4	AGRST/CFHILMVWY/DEKNPQ/X
	3,10	ACFILMVWY/DEKPQR/GHNST/X
	6	AGNRST/CFHILMVWY/DEKPQ/X
	9	AGNRST/CFHILMVWY/DEKPQX
